# Intraventricular Pressure in Non-communicating Hydrocephalus Patients Before Endoscopic Third Ventriculostomy

**DOI:** 10.1515/med-2019-0107

**Published:** 2019-11-29

**Authors:** Werner Tiefenthaler, Johannes Burtscher, Patrizia L. Moser, Ingo H. Lorenz, Christian Kolbitsch

**Affiliations:** 1 Department of Anaesthesia and Intensive Care Medicine, Innsbruck Medical University (MUI), Anichstrasse 35, A-6020 Innsbruck, Austria; 2 Department of Anaesthesia, Medalp Sportclinic, Imst, Austria; 3 Department of Neurosurgery Landesklinikum Wiener, Neustadt, Austria; 4 Department of Pathology, Innpath GmbH, Innsbruck, Austria

**Keywords:** Hypercapnia, Intraventricular pressure, Endoscopic third ventriculostomy

## Abstract

**Background:**

In patients with non-communicating hydrocephalus impairment of cerebral compliance can occur pre- but also intraoperatively.

**Methodology:**

In such patients (n = 6) undergoing endoscopic third ventriculostomy (ETV), the present study aimed to investigate the effect of ETCO2 (e.g 40 mmHg and 60 mmHg) and positive end-expiratory pressure (PEEP) (e.g. 6 cm and 12 cm H2O) on intraventricular pressure (IVP).

**Findings:**

Before but not after ETV, hypercapnia in contrast to PEEP increased IVP

**(before ETV:**

(PEEP-6/ ETCO2-40: 2.6 ± 2.4 mmHg) vs. (PEEP-6/ ETCO2-60: 12 ± 6.4 mmHg*); (PEEP-12/ ETCO2-40: 4.2 ± 4.1 mmHg) vs. (PEEP-12/ ETCO2-60: 13.7 ± 7.6 mmHg*), * significant, P ≤ 0.05;

**after ETV:**

(PEEP-6/ ETCO2-40: 2.0 ± 1.2 mmHg) vs. (PEEP-6/ ETCO2-60: 4.4 ± 3.1 mmHg); (PEEP-12/ ETCO2-40: 1.6 ± 1.3 mmHg) vs. (PEEP-12/ ETCO2-60: 6.6 ± 2.6 mmHg), * significant, P ≤ 0.05).

**Conclusion:**

Patients with non-communicating hydrocephalus showed that hypercapnia but not PEEP increases significantly IVP before but not after ETV.

## 1 Introduction

Cerebral compliance is typically impaired in patients with symptomatic, non-communicating hydrocephalus. To create a near physiological communication between the third ventricle and the basal subarachnoidal spaces endoscopic third ventriculostomy (ETV) has become the treatment of choice for these patients [6,7,9]. The effect of positive end-expiratory pressure (PEEP) on cerebral compliance in these patients and hence the use of PEEP in the respiratory management of these patients, and in general of patients with impaired cerebral compliance, is still a matter of controversy [4, 20]. The present study therefore investigated during concomitant normo- or hypercapnia the effects of several levels of PEEP on intraventricular pressure (IVP) in patients before and after ETV for symptomatic hydrocephalus.

## 2 Methods

Following approval by the local University Ethics Committee and written informed consent, patients (n = 6; ASA physical status I-III) scheduled for ventriculostomic therapy of symptomatic, obstructive third-ventricular hydrocephalus were enrolled in this pilot study.

All patients were premedicated with midazolam (0.1 mg/ kg, p.o.). A bolus of propofol (2 – 3 mg/ kg) and remifentanil (0.2 – 0.4 mcg/ kg / min) was given to induce anesthesia. An additional remifentanil bolus (3 - 5 mcg/kg) facilitated intubation of the trachea [10]. We used tracheal tubes with high-volume, low-pressure cuffs, 7.5mm internal diameter for women and 8.5mm internal diameter for men (Mallinckrodt Inc., St Louis, MO, USA). The cuff was inflated with air, and cuff pressure was monitored and maintained at 20mbar throughout the procedure. To maintain anesthesia propofol (5 mg/ kg/ h) and remifentanil (0.2 – 0.4 mcg/ kg / min) were infused. Controlled ventilation was adjusted to end-tidal CO2 of 40 mmHg. The right radial artery was cannulated (20G catheter) for beat-to-beat blood pressure monitoring.

The patient was in a supine position with the neck slightly flexed. ETV was performed as previously described [8]. A rod lens ventriculoscope (Wolf, Knittlingen, Germany) was used with (had) an outer diameter of 5.8 x 4.8 mm, equiped with a 2.3 mm optical probe and four channels for suction, irrigation and instrument insertion. Blunt perforation of the third ventricular floor was performed in each patient with a 4 Fr. balloon catheter. A ventricular pressure transducer was placed and connected to the monitor (AS/3 Monitor, Datex-Ohmeda, Helsinki, Finland). At baseline intraventricular pressure (IVP) in the third ventricle was recorded at each of four different respiratory settings (e.g. (1) PEEP (6 cm H2O)/ ETCO2 (40 mmHg), (2) PEEP(6 cm H2O)/ ETCO2 (60 mmHg), (3) PEEP(12 cm H2O)/ ETCO2 (40 mmHg), (4) PEEP(12 cm H2O)/ ETCO2 (60 mmHg)). For inducing hypercapnia respiratory settings where chosen with low tidal volume (5-6ml/kg) and/or a low respiratory rate to achieve the desired ETCO2. A 10-minute equlibration period was allowed at each setting before taking the IVP reading. Following ventriculostomy IVP measurements, as described above, were repeated.

Heart rate (HR) and invasive mean arterial blood pressure (IMAP) were monitored intraoperatively.

## 3 Statistical Analysis

Pilot study with a sample size of six patients. Data are presented as mean ± SD. Data were tested for normal distribution using the Kolmogorov-Smirnov Test. Analysis of variance (ANOVA) for repeated measurements was used to compare the IVP values during the four respiratory settings established.

The statistical computer package SPSS ® 11.0.1 for Windows was used for statistical analysis.

## 4 Results

All enrolled patients (n = 6; female (5)/ male (1); age: 52 ± 18 years; weight: 74 ± 11 kg; height: 170 ± 9 cm) completed the study without complication. Technical problems prevented measurement of IVP after ventriculostomy (ETV) in patient No. 6.

Before ETV, increasing PEEP from 6 cm to 12 cm H2O at a constant ETCO2 (e.g. 40 mmHg or 60 mmHg) had no effect on IVP (Table 1). In contrast, increasing ETCO2 from 40 mmHg to 60 mmHg at a constant PEEP (e.g. 6 cm or 12 cm H2O) significantly increased IVP (Table 1). Similarly, IVP increased when PEEP and ETCO2 increased simultaneously but also when PEEP decreased and ETCO2 increased (Table 1).

**Table 1:** Summarizes intraventricular pressure (IVP, mmHg) recordings at 6 cm (6 PEEP) and 12 cm H2O (12 PEEP) positive end-expiratory pressure (PEEP) during normocapnia (40 ETCO2) and hypercapnia (60 ETCO2) before and after endoscopic third ventriculostomy (ETV) in six patients. ^1^ significant to 6 PEEP, 40 ETCO2; P ≤ 0.05 ^2^ significant to 12 PEEP, 40 ETCO2; P ≤ 0.05 ^3^ significant to 6 PEEP, 60 ETCO2; P ≤ 0.05

	Patient	6 PEEP 40 ETCO_2_	6 PEEP 60 ETCO_2_	12 PEEP 40 ETCO_2_	12 PEEP 60 ETCO_2_
IVP (mmHg) before ETV	IVP Patient 1	0	8	2	5
	IVP Patient 2	6	12	7	21
	IVP Patient 3	4	23	11	23
	IVP Patient 4	4	9	3	11
	IVP Patient 5	2	15	2	16
	IVP Patient 6	0	5	0	6
	**mean** ± **SD**	**2.6 ± 2.4**	**12 ± 6.4^1^**	**4.2 ± 4.1^3^**	**13.7 ± 7.6 ^1,2^**
IVP (mmHg) after ETV	IVP Patient 1	2	0	1	6
	IVP Patient 2	3	6	3	6
	IVP Patient 3	2	3	0	4
	IVP Patient 4	3	5	3	6
	IVP Patient 5	0	8	1	11
	IVP Patient 6	-	-	-	-
	**mean ± SD**	**2 ± 1.2**	**4.4 ± 3.1**	**1.6 ± 1.3**	**6.6 ± 2.6**

Changes in PEEP, ETCO2 or both following ETV had no effect on IVP (Table 1).

Hemodynamic parameters remained stable throughout the whole procedure.

## 5 Discussion

In patients with symptomatic, non-communicating hydrocephalus an increase in PEEP (e.g. from 6 cm to 12 cm H2O) had no effect on IVP during normo- or hypercapnia. An increase in ETCO2 (e.g. from 40 mmHg to 60 mmHg), however, increased IVP at 6 and 12 cm H2O PEEP.

A previous study in young, healthy volunteers assessed the effect of increased mean airway pressure (e.g. 12 cm H2O) on cerebrospinal fluid (CSF) drainage from the third to the fourth cerebral ventricle by measuring systolic cerebrospinal fluid peak velocity (CSFVPeak) in the aqueduct of Sylvius [12]. The found decrease in CSFVPeak at 12 cm H2O continuous positive airway pressure (CPAP) was attributed to intrathoracic transmission of positive airway pressure to the spinal CSF. The resulting increase in spinal cerebrospinal fluid pressure (Pcsf) again increased outflow resistance for systolic craniocaudal CSF displacement. Consequently, systolic CSFV-Peak decreased in the aqueduct of Sylvius [12]. In the present study’s patients suffering from non-communicating hydrocephalus, the aqueduct of Sylvius was occluded. Any increase in mean airway pressure (e.g. PEEP from 6 to 12 cm H2O) can via the intrathoracic route increase the Pcsf spinally but also supraspinally and can increase the cerebral blood volume CBV) by impairing cerebrovenous return [13]. Given the significance of both effects, compression of the cerebral ventricles is likely to occur and the intraventricular pressure (IVP) should significantly rise in the third ventricle, especially since compensatory CSF drainage to the fourth ventricle is not possible. Interestingly enough, the present study’s IVP did not rise when increasing PEEP from 6 to 12 cm H2O during normo- or hypercapnia.

Changes in arterial carbon dioxide partial pressure (paCO2), which correlate well with ETCO2 [1,2] cause changes in CBV [19], thereby increasing to a certain extent the pressure on the cerebral ventricles. A consequent IVP rise was previously shown indirectly in cerebrally healthy patients when hypercapnia (e.g. ETCO2 = 60 mmHg, PEEP = 5 cm H2O) increased CSFVPeak in the aqueduct of Sylvius [11]. Similarly, the present study’s hypercapnia increased IVP at PEEP 6 cm H2O in patients with non-communicating hydrocephalus. A comparable increase in IVP was also found in these patients when establishing hypercapnia at an increased mean airway pressure, namely PEEP 12 cmH2O.

In summary an increase in CBV, e.g. by hypercapnia, much more so than an increase in Pcsf, e.g. by increasing mean airway pressure, is clearly seen to increase IVP due to the increased pressure on the cerebral ventricles. These findings are consistent with those of others, who reported a PEEP of 15 – 20 cm H2O to not significantly increase intracranial pressure (ICP), for example in head trauma patients [15], whereas an increase in paCO2 dramatically increased ICP [3].

Immediately following ETV the present study’s IVP measurements taken during normo- and hypercapnia were repeated at 6 and 12 cm H2O PEEP. Similar to the readings before ETV, increasing PEEP from 6 to 12 cm H2O had no effect on IVP. Surprisingly, in contrast to before ETV, hypercapnia now did not increase IVP, neither at 6 cm nor at 12 cm H2O PEEP. Obviously, the ventriculostoma allowed sufficient CSF drainage from the third ventricle, so that hypercapnia-induced increases in CBV compressed the third ventricle without concomitantly increasing IVP. Since CSF drainage by way of a patent aqueduct of Sylvius was previously shown to not be able to compensate hypercapnia-induced compression of the third ventricle (e.g. CSFVPeak increased) [11] it is logical that the diameter of the ventriculostoma (approx. 5 mm) must be larger the normal diameter (e.g. 2 - 3 mm [18] of the aqueduct of Sylvius.

Propofol and remifentanil were used to induce and maintain anesthesia in our patients. Any confounding effect of either anesthetic agent on our results can largely be excluded for two reasons. Firstly, propofol but also remifentanil are known to preserve cerebral autoregulation[14,16,17] and CO2-reactivity, [14,16,17]. Secondly, both agents were always simultaneously present during the various combinations of ETCO2 and PEEP, so that a potential influence on the IVP readings should be the same at all measuring times.

From a practical, clinical point of view it is important to emphasize that hypercapnia should be strictly avoided in patients with symptomatic, non-communicating hydrocephalus, especially before successful ETV, whereas an increase in mean airway pressure is well tolerable.

The present study’s patients showed stable hemodynamics throughout the procedure. It is, however, noteworthy that in about 7% of ETV procedures a Cushing reflex can occur in response to increased ICP [21]. Especially abrupt increases in ICP initiate the Cushing triad of apnoea, systemic hypertension and initial tachycardia, which deteriorates to bradycardia if ICP elevation persists [5]. It is above all compression of the hypothalamus that in this context causes the tachycardia. The fact that the present study’s hypercapnia increased IVP without initiating the Cushing triad is due either to insufficient IVP increase or to the small number of patients (e.g. n = 6) enrolled combined with the overall low incidence (e.g. approx. 7%) of Cushing reflex during ETV procedures.

In conclusion, we here show that in patients with non-communicating hydrocephalus hypercapnia but not PEEP significantly increased IVP before but not after ETV.
